# Lipid Nanoparticle Acts as a Potential Adjuvant for Influenza Split Vaccine without Inducing Inflammatory Responses

**DOI:** 10.3390/vaccines8030433

**Published:** 2020-08-03

**Authors:** Seiki Shirai, Atsushi Kawai, Meito Shibuya, Lisa Munakata, Daiki Omata, Ryo Suzuki, Yasuo Yoshioka

**Affiliations:** 1 Laboratory of Nano-design for Innovative Drug Development, Graduate School of Pharmaceutical Sciences, Osaka University, 1-6 Yamadaoka, Suita, Osaka 565-0871, Japan; s-shirai@biken.osaka-u.ac.jp (S.S.); kawai-a@phs.osaka-u.ac.jp (A.K.); shibuya-m@phs.osaka-u.ac.jp (M.S.); 2 Vaccine Creation Group, BIKEN Innovative Vaccine Research Alliance Laboratories, Institute for Open and Transdisciplinary Research Initiatives, Osaka University, 3-1 Yamadaoka, Suita, Osaka 565-0871, Japan; 3 Vaccine Creation Group, BIKEN Innovative Vaccine Research Alliance Laboratories, Research Institute for Microbial Diseases, Osaka University, 3-1 Yamadaoka, Suita, Osaka 565-0871, Japan; 4 Laboratory of Drug and Gene Delivery Research, Faculty of Pharma-Science, Teikyo University, 2-11-1 Kaga, Itabashi, Tokyo 173-8605, Japan; munakata@pharm.teikyo-u.ac.jp (L.M.); omata@pharm.teikyo-u.ac.jp (D.O.); r-suzuki@pharm.teikyo-u.ac.jp (R.S.); 5 Vaccine Creation Group, BIKEN Innovative Vaccine Research Alliance Laboratories, The Research Foundation for Microbial Diseases, Osaka University, 3-1 Yamadaoka, Suita, Osaka 565-0871, Japan; 6 Global Center for Medical Engineering and Informatics, Osaka University, 3-1 Yamadaoka, Suita, Osaka 565-0871, Japan

**Keywords:** adjuvant, dendritic cell, inflammation, influenza virus, lipid nanoparticle, vaccine

## Abstract

Vaccination is a critical and reliable strategy for controlling the spread of influenza viruses in populations. Conventional seasonal split vaccines (SVs) for influenza evoke weaker immune responses than other types of vaccines, such as inactivated whole-virion vaccines, although SVs are highly safe compared to other types. Here, we assessed the potential of the lipid nanoparticle (LNP) we developed as an adjuvant for conventional influenza SV as an antigen in mice. The LNP did not induce the production of cytokines such as interleukin-6 (IL-6) and IL-12 p40 by dendritic cells or the expression of co-stimulatory molecules on these cells in vitro. In contrast, an SV adjuvanted with LNP improved SV-specific IgG1 and IgG2 responses and the Th1 response compared to the SV alone in mice. In addition, SV adjuvanted with an LNP gave superior protection against the influenza virus challenge over the SV alone and was as effective as SV adjuvanted with aluminum salts in mice. The LNP did not provoke inflammatory responses such as inflammatory cytokine production and inflammatory immune cell infiltration in mice, whereas aluminum salts induced inflammatory responses. These results suggest the potential of the LNP as an adjuvant without inflammatory responses for influenza SVs. Our strategy should be useful for developing influenza vaccines with enhanced efficacy and safety.

## 1. Introduction

Influenza viruses remain of serious health concern worldwide, particularly among the very young, the elderly, and the chronically ill; annual epidemics caused by influenza viruses affect 5–15% of the global population [1]. Vaccination is one of the most critical and reliable strategies for preventing severe illness and controlling the spread of influenza viruses at the population level. Current vaccines for influenza virus include attenuated live vaccines, inactivated whole-virion vaccines, and split vaccines (SVs) [2,3]. These vaccines predominantly induce neutralizing antibodies to the receptor-binding site located on hemagglutinin (HA), which is a major virus-surface protein that mediates virus entry into susceptible cells [1]. Each type of vaccine has advantages and disadvantages. For example, although inactivated whole-virion vaccines can generate strong adaptive immunity, they can cause inflammatory responses that might induce adverse reactions [4]. In contrast, although SVs alone evoke weaker B-cell and T-cell responses than inactivated whole-virion vaccines, SVs are very safe compared to whole-virion vaccines [1,4]. One means for improving the efficacy of SVs is to choose an appropriate adjuvant [5].

In accordance with their mechanisms of action, adjuvants are commonly classified as immunomodulatory adjuvant molecules, delivery vehicles, and a combination of both. Immunomodulatory adjuvant molecules mainly activate innate immune responses through pattern recognition receptors such as toll-like receptors (TLRs) [6,7]. A wide variety of natural and synthetic immunomodulatory adjuvant molecules have been developed in preclinical and clinical studies [7]. For example, alum (aluminum salts) is one of the most reliable adjuvants, and many vaccines formulated with alum have been used throughout the world [7]. In addition, oligodeoxynucleotides (ODNs) with unmethylated cytosine–phosphate–guanine (CpG) motifs (CpG ODNs)—TLR9 agonists—are among the most effective adjuvants for inducing not only antibody responses but also Th1-type immune responses and CD8^+^ T-cell responses [8]. A specific type of CpG ODN has already been approved for clinical use as a hepatitis B virus vaccine adjuvant [9]. However, systemic inflammation in response to the presence of immunomodulatory adjuvant molecules is typically responsible for adjuvant-dependent adverse reactions [10,11]. Therefore, several types of delivery vehicles for immunomodulatory adjuvant molecules have been developed to mitigate systemic inflammation and enhance potent adjuvant effects [12,13,14].

In addition to adjuvants that act as delivery vehicles for immunomodulatory adjuvant molecules, several functionalized nanoparticles, including polymer- and lipid-based nanoparticles, have emerged as adjuvants that act as efficient vehicles for the delivery of vaccine antigens to targeted immune cells, such as dendritic cells (DCs) [12,13,15]. Among these nanoparticles, lipid nanoparticles (LNPs)—typically composed of an ionizable lipid, cholesterol, lipid conjugated with polyethylene glycol, and a helper lipid—have recently attracted much attention as novel, lipid-based antigen-delivery vehicles [16,17]. LNPs are generally widely used as delivery systems for DNA- or RNA-based medicines because LNPs with highly efficient encapsulation of DNA- or RNA-based medicines can readily be produced by using a microfluidic mixer [16,17]. For vaccine application, LNPs containing mRNA coding for protein antigens have been used to induce strong immune responses, such as antigen-specific antibody responses and T-cell responses in mice and rhesus macaques [18,19]. In addition, there is increasing evidence of the usefulness of LNPs as efficient antigen-delivery vehicles [20,21,22,23,24,25]. For example, by using protein antigens from dengue virus and hepatitis B virus, it has been shown that specific LNPs can enhance co-administered antigen-specific antibody responses, CD4^+^ T-cell responses, and CD8^+^ T-cell responses in mice, guinea pigs, and non-human primates without the addition of immunomodulatory adjuvant molecules [23,24,25], although the precise mechanism of adjuvanticity of specific LNPs remains unclear. However, the potential of LNPs as delivery systems for SVs generated from influenza virus remains unclear.

We have demonstrated the potential of the LNP we developed as a CpG ODN delivery vehicle [26,27]. We have already demonstrated that LNP containing CpG ODN (LNP-CpG) has stronger immune-activating activity in vitro and in vivo than CpG ODN [26,27]. In addition, LNP-CpG induces stronger antigen-specific antibody responses than CpG ODN in mice vaccinated with SV generated from influenza virus [27]. However, the usefulness of our LNP as an antigen-delivery vehicle remains unclear.

Here, we showed the usefulness of our LNP as an adjuvant for an SV generated from influenza virus as an antigen. Compared to the SV alone, SV given with the LNP improved antigen-specific IgG1 and IgG2 responses and the Th1 response in mice. In addition, SV adjuvanted with the LNP showed superior protective effects against influenza virus challenge in mice, and this protection was as effective as that obtained with SV adjuvanted with alum. The LNP did not induce inflammatory responses in mice, whereas alum induced inflammatory responses. These results suggest the potential of the LNP as a superior adjuvant for influenza vaccines.

## 2. Materials and Methods

### 2.1. Reagents

1,2-dioleoyl-3-trimethylammonium-propane was purchased from Lipoid GmbH (Ludwigshafen, Germany). 1,2-dipalmitoyl-sn-glycero-3-phosphocholine and N-(carbonyl-methoxypolyethyleneglycol 2000)-1,2-distearoyl-sn-glycero-3-phosphoethanolamine were purchased from NOF Corporation (Tokyo, Japan). Cholesterol was purchased from Fujifilm Wako Pure Chemical Corporation, Ltd. (Osaka, Japan). D-type CpG ODN (CpG ODN: 5′-ggtgcatcgatgcagggggg-3′) was purchased from GeneDesign (Osaka, Japan). Horseradish-peroxidase-conjugated goat anti-mouse IgG was purchased from Merck Millipore (Darmstadt, Germany). Horseradish-peroxidase-conjugated goat anti-mouse IgG1, IgG2b, and IgG2c were purchased from SouthernBiotech (Birmingham, AL, USA). Alhydrogel adjuvant 2%, as alum, was purchased from InvivoGen (San Diego, CA, USA). Ether-treated, HA-antigen-enriched, virion-free SV from H1N1 influenza A virus (strain: A/California/7/2009 (Cal7)) was kindly provided by Dr. Yasuyuki Gomi of the Research Foundation for Microbial Diseases of Osaka University, Osaka, Japan. The SV contained 570 μg of HA in 1000 μg of SV. H1N1 influenza A virus (strain: Cal7) was kindly provided by Dr. Hideki Asanuma of the National Institute of Infectious Diseases, Tokyo, Japan.

### 2.2. Mice

Male C57BL/6J mice were purchased from SLC (Kyoto, Japan). Mice were used at 6–7 weeks of age and were housed in a room with a 12:12-h light:dark cycle (lights on, 8:00 a.m.; lights off, 8:00 p.m.). They had unrestricted access to food and water. All animal experiments were performed in accordance with Osaka University’s institutional guidelines for the ethical treatment of animals (protocol number H26-11-0).

### 2.3. Synthesis of LNP and LNP-CpG

The LNP was prepared by using a NanoAssemblr Benchtop nanoparticle prototyping system (Precision NanoSystems Inc., Vancouver, BC, Canada), which could mediate bottom-up self-assembly for nanoparticle synthesis with microfluidic mixing technology. 1,2-dioleoyl-3-trimethylammonium-propane, 1,2-dipalmitoyl-sn-glycero-3-phosphocholine, cholesterol, and N-(carbonyl-methoxypolyethyleneglycol 2000)-1,2-distearoyl-sn-glycero-3-phosphoethanolamine were, respectively, dissolved in ethanol at a molar ratio of 50:19.5:30:0.5. The lipid solution (10 mg/mL) in ethanol and a 25 mM acetate buffer at pH 4.0 were injected into the microfluidic mixer at a 1:3 volume, respectively, and a combined final flow rate of 12 mL/min (3.0 mL/min ethanol and 9.0 mL/min aqueous). The mixtures were immediately dialyzed (300-kD MWCO dialysis tubing, Repligen Corporation, Waltham, MA, USA) against a 5% glucose solution to remove the ethanol. The prepared LNP was filtered through a 0.22-μm PVDF filter (Merck KGaA, Darmstadt, Germany). An LNP containing CpG ODN (LNP-CpG) was also prepared by using NanoAssemblr Benchtop, as described previously [26,27]. All LNP preparation work was performed at room temperature. We used a 5% glucose solution as a control buffer in all experiments, because this same solution was used for the LNP and LNP-CpG. The size distributions of LNP and LNP-CpG were measured by using dynamic light scattering (Zetasizer Nano-ZS, Malvern Panalytical Ltd., Malvern, UK).

### 2.4. Production and Purification of Recombinant HA and Neuraminidase Proteins

The amino acid sequence for the HA used here was derived from Cal7 (GenBank accession number: ACV82259.1). The amino acid sequences for the neuraminidase (NA) that we used were derived from Cal7 (GenBank accession number: MN596847.1). The human codon-optimized cDNA of the ectodomain of HA with a C-terminal hexahistidine tag (His-tag) and of NA with an N-terminal His-tag were cloned into a pcDNA3.1 expression plasmid (Thermo Fisher Scientific, Hampton, NH, USA). The codon-optimized foldon trimerization domain sequence was inserted at the C-terminal of HA, and the tetrabrachion tetramerization domain sequence was inserted at the N-terminal of NA, as previously described [28,29]. All secreted soluble recombinant HA (rHA) and recombinant NA (rNA) were expressed by using an Expi293 Expression System (Thermo Fisher Scientific) in accordance with the manufacturer’s instructions. The rHA and rNA in the supernatant were then purified by using an AKTA Explorer chromatography system with an Ni-Sepharose HisTrap FF column (GE Healthcare, Diegem, Belgium) and a Superose 6 Increase 10/300 GL column (GE Healthcare).

### 2.5. Preparation and Stimulation of Mouse Bone-Marrow-Derived DCs

To generate bone-marrow-derived DCs, we isolated bone marrow cells from the femurs of C57BL/6J mice and cultured the cells at 37 °C for 7 days with 100 ng/mL human Fms-related tyrosine kinase 3 ligand (PeproTech, Rocky Hill, NJ, USA). Cells were seeded at a density of 1 × 10^5^ cells/well in a 96-well flat-bottomed culture plate (Nunc, Roskilde, Denmark) and were cultured in complete RPMI medium (RPMI 1640 supplemented with 10 vol.% fetal calf serum, penicillin, and streptomycin). These cells were stimulated with an LNP (13.3 μg lipid/mL or 4.4 μg lipid/mL) or LNP-CpG (13.3 μg lipid with 1.0 μg CpG ODN/mL or 4.4 μg lipid with 0.33 μg CpG ODN/mL) at 37 °C for 24 h. Supernatants were subjected to ELISA to determine the levels of interleukin (IL)-6 (BioLegend, San Diego, CA, USA), IL-12 p40 (BioLegend), tumor necrosis factor (TNF)-α (BioLegend), and interferon (IFN)-α (InvivoGen) in accordance with the manufacturers’ instructions. To check the levels of co-stimulatory molecules on the DCs, we incubated the cells with an anti-mouse CD16/CD32 antibody (clone: 93, BioLegend), an APC/Cy7 anti-CD11c antibody (clone: N418, BioLegend), a fluorescein isothiocyanate (FITC) anti-CD80 antibody (clone: 16–10A1, BioLegend), and a phycoerythrin (PE) anti-CD86 antibody (clone: GL-1, BioLegend). Then, the cells were analyzed by means of flow cytometry (NovoCyte Flow Cytometer, ACEA Biosciences, San Diego, CA, USA).

### 2.6. Immunization

Mice were subcutaneously immunized at the base of the tail once (on day 0) or twice (on days 0 and 21) by using SV (0.5 μg/mouse) from Cal7 without or with the LNP (130 μg lipid/mouse), LNP-CpG (130 μg lipid with 10 μg CpG ODN/mouse), or alum (50 μg/mouse). On day 14 (for the one-immunization protocol) or day 28 (for the two-immunizations protocol), we obtained plasma samples, and the levels of SV-specific antibodies in the plasma were determined by ELISA. For immunization with rHA, mice were subcutaneously immunized at the base of the tail on days 0 and 21 by using rHA (1 μg/mouse) from Cal7 without or with the LNP (130 μg lipid/mouse), LNP-CpG (130 μg lipid with 10 μg CpG ODN/mouse), or alum (50 μg/mouse). On day 28, we obtained plasma samples, and the levels of rHA-specific antibodies in the plasma were determined by ELISA. To detect SV-specific total IgG, IgG1, IgG2b, and IgG2c (Figures 2 and 3), rHA- (Figure 6, Supplementary Figure S2), or rNA- (Supplementary Figure S2) specific total IgG levels, ELISA plates (Corning, Corning, NY, USA) were coated overnight at 4 °C with the SV (10 μg/mL) in phosphate-buffered saline (PBS), rHA (1 μg/mL) in a carbonate buffer, or rNA (1 μg/mL) in a carbonate buffer. The coated plates were then incubated with 1% Block Ace (DS Pharma Biomedical, Osaka, Japan) for 2 h at room temperature. Plasma samples were diluted with 0.4% Block Ace, and these dilutions were added to the antigen-coated plates. After incubation with plasma for 2 h at room temperature, the coated plates were incubated with a horseradish-peroxidase-conjugated goat anti-mouse IgG, IgG1, IgG2b, or IgG2c solution for 1 h at room temperature. After the incubation, the color reaction was developed with tetramethyl benzidine (Nacalai Tesque, Kyoto, Japan), stopped with 2 N H_2_SO_4_, and measured at OD_450–570_ on a microplate reader (Power Wave HT, BioTek, Winooski, VT, USA).

### 2.7. Neutralization Assay

Plasma samples were incubated with the RDE (receptor destroying enzyme) (II) (Denka Seiken, Tokyo, Japan) for 18 h at 37 °C and then heated at 56 °C for 1 h to deactivate the enzyme. Mixtures of two-fold serially diluted plasma samples and influenza virus with final concentrations of 100  × TCID_50_ virus per mixture were incubated at 37 °C for 30 min. The mixtures were subsequently added to Madin–Darby canine kidney cells and incubated at 37 °C for 3 days. The cells were then fixed with 4% paraformaldehyde at room temperature for 10 min. The cells were stained with 0.1% amido black (Nacalai Tesque) in an acetic acid solution at room temperature for 30 min. After the cells had been washed with water, 0.1 N NaOH was added and the OD_630_ was measured on a microplate reader.

### 2.8. Enzyme-Linked Lectin Assay (ELLA) to Determine Neuraminidase Inhibition (NI)

ELISA plates were coated overnight at 4 °C with 25 μg/mL fetuin (Sigma-Aldrich, St Louis, MO, USA) in a carbonate buffer. Serially diluted plasma samples were mixed with 200 ng/mL rNA and incubated for 2 h at 37 °C in separate 96-well plates, and these mixtures were added to the fetuin-coated plates. After incubating at 37 °C for 16 h, a peanut agglutinin–horseradish peroxidase conjugate (Sigma-Aldrich) was added. After 1 h of incubation at room temperature, the color reaction was developed with tetramethyl benzidine, stopped with 2 N H_2_SO_4_, and measured at OD_450_ on a microplate reader.

### 2.9. Cytokine Production by Splenocytes after Immunization

Mice were subcutaneously immunized at the base of the tail on days 0 and 21 by using the SV (0.5 μg/mouse) or rHA (1 μg/mouse) without or with the LNP (130 μg lipid/mouse), LNP-CpG (130 μg lipid with 10 μg CpG ODN/mouse), or alum (50 μg/mouse). On day 28, spleens were collected and splenocytes were prepared to determine cytokine production. Splenocytes (1 × 10^6^ cells) were added to the wells of a 96-well plate. They were then stimulated with the SV (10 μg/mL) or rHA (10 μg/mL) for 1 or 5 days at 37 °C, or they were left unstimulated. After the incubation, the concentrations of IL-2, IL-13, and IFN-γ in the supernatants were analyzed by ELISA (IL-2 and IFN-γ: BioLegend; IL-13: R&D Systems, Minneapolis, MN, USA) in accordance with the manufacturers’ instructions.

### 2.10. Influenza Virus Challenge after Immunization

After immunization with the SV (0.5 μg/mouse) from Cal7 without or with the LNP (130 μg lipid/mouse), LNP-CpG (130 μg lipid with 10 μg CpG ODN/mouse), or alum (50 μg/mouse) on day 0, anesthetized mice were intranasally challenged with 3 × 10^4^ TCID_50_ of Cal7 in 30 μL of PBS on day 21. The body weights and survival rates of challenged mice were monitored. The humane endpoint was set at 25% body weight loss relative to the initial body weight at the time of infection.

### 2.11. Inflammation Assay

To assess the recruitment of immune cells and inflammatory cytokine release, mice were intraperitoneally treated with the SV (0.5 μg/mouse) alone, SV plus LNP (130 μg lipid/mouse), or the SV plus alum (50 μg/mouse). After 4 or 20 h, mice were euthanized under anesthesia, and a 1 mL PBS buffer was injected into the peritoneal cavity, the area was massaged 30 times, and 0.7 mL of lavage fluid was collected. The lavage fluid was centrifuged at 600× *g* for 5 min, and cell pellets were collected for flow cytometric analysis. To separate the various subsets of immune cells in the lavage fluid, prepared cells were incubated with the anti-mouse CD16/CD32 antibody (clone: 93, BioLegend), PE anti-mouse F4/80 (clone: BM8, BioLegend), Brilliant Violet 785 anti-mouse CD11b (clone: M1/70, BioLegend), PE-Cy7 anti-mouse Ly-6C (clone: HK1.4, BioLegend), or FITC anti-mouse Ly-6G (clone: 1A8, BioLegend) for flow cytometry. The concentrations of IL-6 and granulocyte-colony stimulating factor (G-CSF) in the supernatants of lavage fluid after centrifugation were analyzed by ELISA in accordance with the manufacturers’ instructions (IL-6: BioLegend; G-CSF: R&D Systems).

### 2.12. Statistical Analyses

Statistical analyses were performed by using Prism (GraphPad Software, San Diego, CA, USA). All data are presented as means with SD. Significant differences were determined by means of Tukey’s test. A *p* value of <0.05 was considered to indicate statistical significance.

## 3. Results

### 3.1. In Contrast to LNP-CpG, LNP Does Not Induce Cytokine Production or Enhance the Expression of Co-Stimulatory Molecules in Mouse DCs

We investigated the usefulness of the LNP as an adjuvant and compared the immune responses induced by the LNP, LNP-CpG, and alum. The LNP and LNP-CpG were prepared by using a microfluidic mixer system, NanoAssemblr, as described previously [26,27]. The hydrodynamic diameter of the LNP was 86.5 nm (polydispersity index: 0.277), and the zeta potential of the LNP was 67.9 ± 12.1 mV. We had already confirmed the physical properties of LNP-CpG: The hydrodynamic diameter of LNP-CpG was 54.0 nm (polydispersity index: 0.157), and the zeta potential of LNP-CpG was 1.12 ± 8.26 mV [26,27].

**Figure 1:**
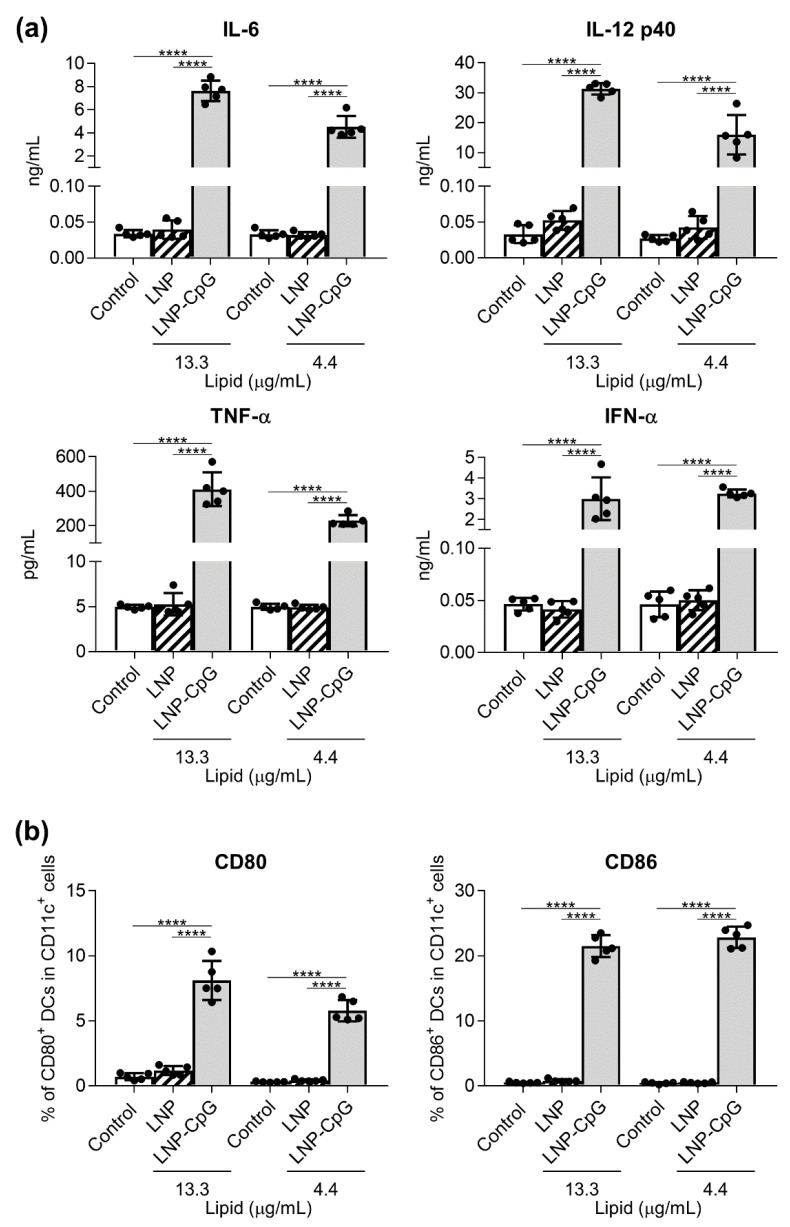
Cytokine production by mouse-derived dendritic cells (DCs) in response to the lipid nanoparticle (LNP) and LNP-cytosine–phosphate–guanine (LNP-CpG) in vitro. Mouse-derived DCs were treated with the LNP (13.3 or 4.4 μg lipid/mL) or LNP-CpG (13.3 μg lipid with 1.0 μg CpG oligodeoxynucleotide (ODN)/mL or 4.4 μg lipid with 0.33 μg CpG ODN/mL) for 24 h in vitro. (**a**) Levels of interleukin (IL)-6, IL-12 p40, tumor necrosis factor (TNF)-α and interferon (IFN)-α in the supernatants were measured by using ELISA. (**b**) Expression levels of CD80 and CD86 on DCs were measured by flow cytometry; percentages of positive DCs are shown. (**a**,**b**) *n* = 5 per group. Data are means ± SD. **** *p* < 0.0001, as indicated by Tukey’s test.

To compare the immune-stimulatory effect of the LNP on DCs with that of LNP-CpG, mouse-derived DCs were treated with the LNP or LNP-CpG in vitro. We examined the concentrations of cytokines in the supernatants (Figure 1a) and the expression levels of co-stimulatory molecules on DCs (Figure 1b). As we previously demonstrated [27], DCs stimulated with LNP-CpG produced significantly greater concentrations of IL-6, IL-12 p40, TNF-α, and IFN-α, and they significantly enhanced the expression of CD80 and CD86 compared to untreated control DCs or DCs stimulated with the LNP. In contrast, we did not observe any cytokine production by, or enhanced expression of CD80 and CD86 on, LNP-treated DCs compared to untreated control DCs. Additionally, we did not observe any increased IL-6 or IL-12 p40 production by H1N1-influenza-A-virus-derived SV-treated DCs or SV-plus-LNP-treated DCs compared to untreated control DCs (Supplementary Figure S1). These data suggest that, unlike LNP-CpG, the LNP does not have immune-stimulatory adjuvant activity in vitro.

### 3.2. LNP Improves Antigen-Specific Antibody Responses and Th1 Responses Induced by SV

**Figure 2:**
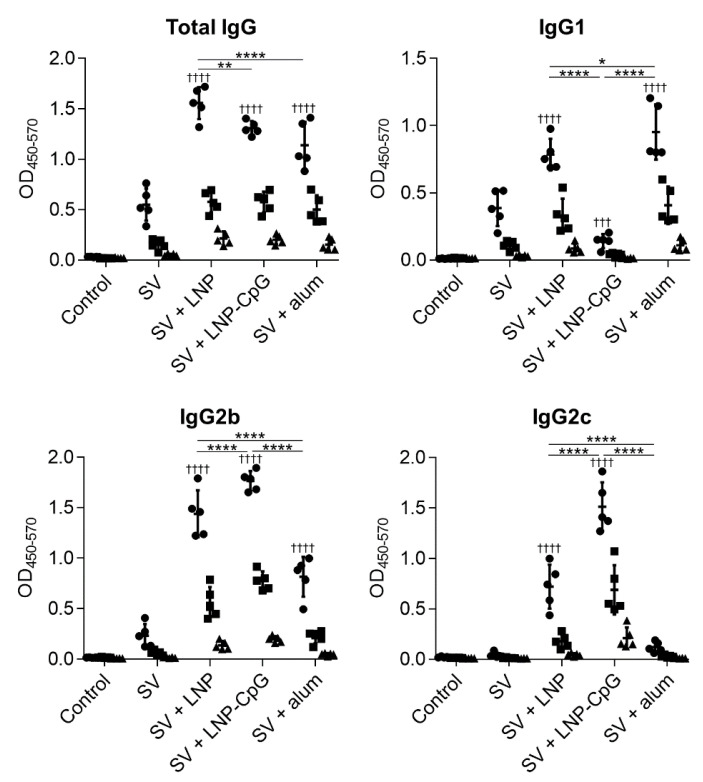
Split vaccine (SV)-specific antibody responses in vivo after first immunization. Mice were subcutaneously immunized with the SV alone, SV plus LNP, SV plus LNP-CpG, or SV plus alum on day 0. On day 14, levels of SV-specific total IgG, IgG1, IgG2b, and IgG2c in plasma were evaluated by using ELISA. We used 800- (●), 4000- (■), and 20,000- (▲) fold diluted plasma samples. *n* = 5 per group. Data are means ± SD. Significant differences were only analyzed in the 800-fold-diluted plasma samples. ^†††^ *p* < 0.001, and ^††††^ *p* < 0.0001 vs. group immunized with the SV alone; * *p* < 0.05, ** *p* < 0.01, and **** *p* < 0.0001, as indicated by Tukey’s test.

To examine the usefulness of the LNP as an adjuvant for a conventional seasonal SV, mice were immunized with the SV alone or SV plus LNP. We used SV plus LNP-CpG (same lipid content as LNP) or SV plus alum as positive controls. The SV from H1N1 influenza A virus (strain: A/California/7/2009 (Cal7)) was used. The plasma levels of SV-specific total IgG, IgG1, IgG2b, and IgG2c antibodies were analyzed by using ELISA at 14 days after the first immunization (Figure 2). Mice immunized with SV plus LNP had significantly higher levels of SV-specific total IgG, IgG1, IgG2b, and IgG2c than those given the SV alone. Mice immunized with SV plus LNP-CpG had significantly lower IgG1 levels than those given the SV alone, and there was no difference in IgG2c level between mice immunized with the SV alone and those given SV plus alum. The levels of SV-specific total IgG, IgG2b, and IgG2c were significantly higher in mice immunized with SV plus LNP than in those immunized with SV plus alum, whereas the level of SV-specific IgG1 in mice immunized with SV plus LNP was significantly lower than that in mice immunized with SV plus alum. Mice immunized with SV plus LNP-CpG had the highest levels of SV-specific IgG2b and IgG2c among all groups, whereas the levels of SV-specific total IgG and IgG1 in mice immunized with SV plus LNP-CpG were significantly lower than those in mice immunized with SV plus LNP.

**Figure 3:**
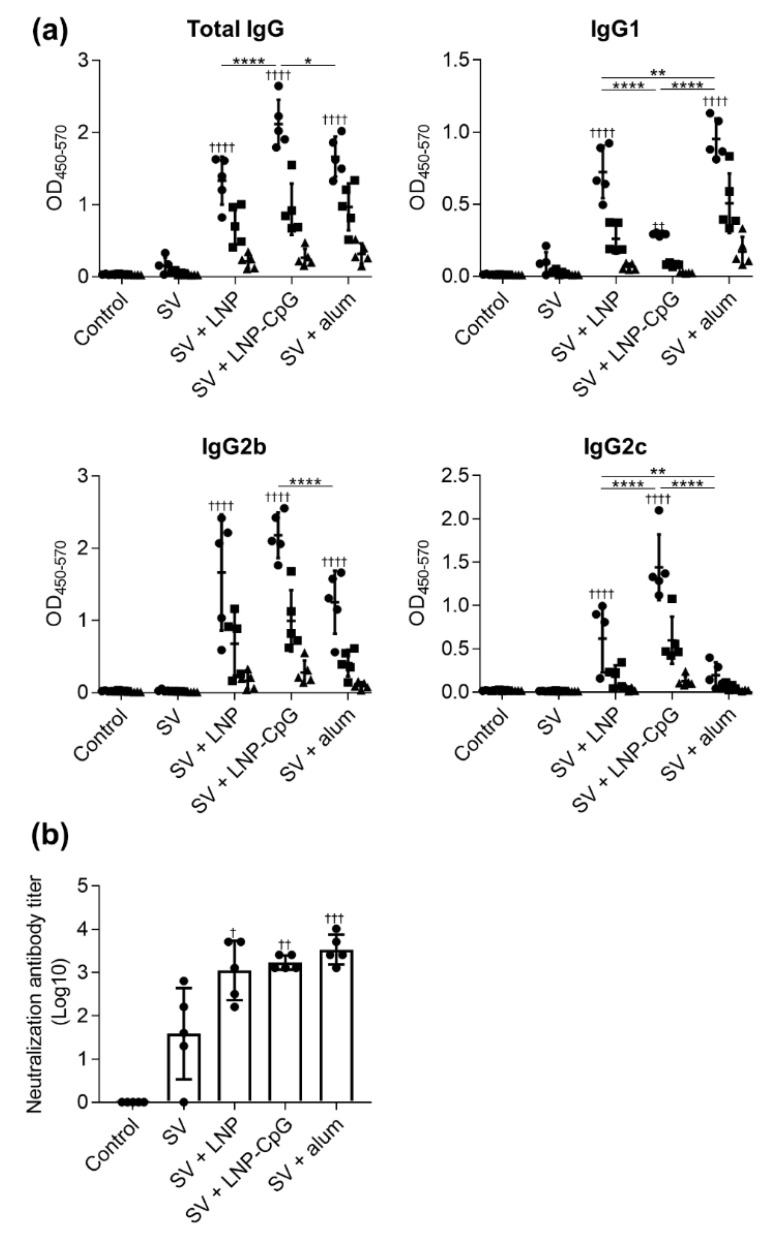
SV-specific antibody responses in vivo after second immunization. Mice were subcutaneously immunized with the SV alone, SV plus LNP, SV plus LNP-CpG, or SV plus alum on days 0 and 21. (**a**) On day 28, levels of SV-specific total IgG, IgG1, IgG2b, and IgG2c in plasma were evaluated by using ELISA. We used 4000- (●), 20,000- (■), and 100,000- (▲) fold diluted plasma samples. (**b**) Neutralization titers in plasma samples against Cal7 were evaluated. (**a**,**b**) *n* = 5 per group. Data are means ± SD. (**a**) Significant differences were only analyzed in the 4000-fold-diluted plasma samples. (**a**,**b**) ^†^ *p* < 0.05, ^††^ *p* < 0.01, ^†††^ *p* < 0.001, and ^††††^ *p* < 0.0001 vs. group immunized with the SV alone; * *p* < 0.05, ** *p* < 0.01, and **** *p* < 0.0001, as indicated by Tukey’s test.

Next, we examined the plasma levels of SV-specific total IgG, IgG1, IgG2b, and IgG2c antibodies at seven days after the second immunization (Figure 3a). The trends in antibody responses were generally similar to those after the first immunization. Mice immunized with SV plus LNP or SV plus LNP-CpG had significantly higher levels of SV-specific total IgG, IgG1, IgG2b, and IgG2c than those given the SV alone. There were no significant differences in SV-specific total IgG and IgG2b between mice given SV plus LNP and those receiving SV plus alum. The level of the SV-specific IgG2c in mice immunized with SV plus LNP was significantly higher than that in mice immunized with SV plus alum, whereas the level of SV-specific IgG1 in mice immunized with SV plus LNP was significantly lower than that in mice immunized with SV plus alum. Mice immunized with SV plus LNP-CpG had the highest levels of SV-specific total IgG and IgG2c among all groups, whereas the level of SV-specific IgG1 in mice immunized with SV plus LNP-CpG was significantly lower than that in mice immunized with SV plus LNP or SV plus alum.

Next, we examined the neutralizing activity of plasma from mice at seven days after the second immunization (Figure 3b). The plasma from mice immunized with SV plus LNP, SV plus LNP-CpG, or SV plus alum had significantly higher neutralizing activity than that from mice given the SV alone. These results suggest that SV plus LNP can enhance the SV-induced neutralization of SV-specific IgG1 and IgG2.

To examine the antigen specificity of plasma from immunized mice, we used ELISA to evaluate the plasma levels of total IgG specific to rHA from Cal7 (Supplementary Figure S2a) and rNA from Cal7 (Supplementary Figure S2b). Consistent with the SV-specific antibody responses, the levels of total IgG specific to rHA and rNA in mice given SV plus LNP were significantly higher than those in mice given the SV alone. There were no differences in rHA-specific total IgG levels among mice immunized with SV plus LNP, SV plus LNP-CpG, and SV plus alum, whereas the level of rNA-specific total IgG was significantly lower in mice immunized with the SV plus LNP than in animals immunized with SV plus LNP-CpG or SV plus alum. In addition, the neuraminidase inhibition (NI) titers in plasma showed the same trend as the levels of rNA-specific total IgG and mice immunized with SV plus LNP showed higher NI titers than mice given the SV alone (Supplementary Figure S2c).

**Figure 4:**
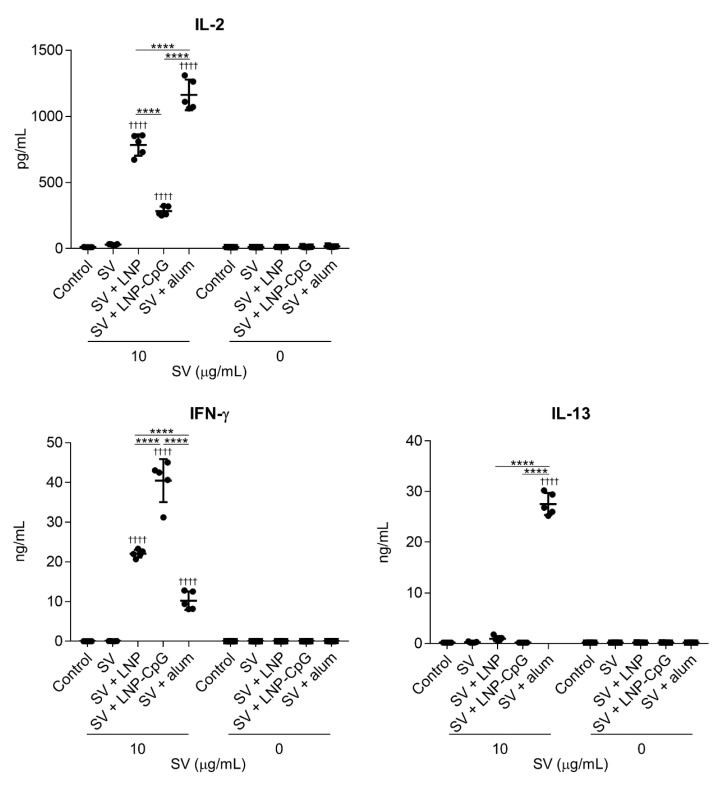
SV-specific T-cell responses in vivo. Mice were subcutaneously immunized with the SV alone, SV plus LNP, SV plus LNP-CpG, or SV plus alum on days 0 and 21. On day 28, splenocytes were cultured in the presence or absence of the SV in vitro. After 1 (for IL-2) or 5 (for IFN-γ and IL-13) days, the levels of IL-2, IFN-γ, and IL-13 were measured by using ELISA. *n* = 5 per group. Data are means ± SD. ^††††^ *p* < 0.0001 vs. group immunized with the SV alone; **** *p* < 0.0001, as indicated by Tukey’s test.

To investigate T-cell responses, splenocytes were recovered from the spleen after the second immunization and stimulated with the SV in vitro, and the levels of IL-2, IFN-γ, and IL-13 in the supernatant were measured by using ELISA (Figure 4). The level of IL-2 in mice immunized with SV plus LNP was significantly higher than those in mice given the SV alone or SV plus LNP-CpG. In addition, the level of IFN-γ in mice immunized with SV plus LNP was significantly higher than those in mice given the SV alone or SV plus alum, whereas mice immunized with SV plus LNP-CpG had the highest level of IFN-γ among all groups. We did not observe significant differences in IL-13 among mice immunized with the SV alone, SV plus LNP, or SV plus LNP-CpG. The levels of IL-2 and IL-13 in mice immunized with SV plus alum were significantly higher than those in mice given the SV alone, SV plus LNP, or SV plus LNP-CpG. These results suggest that the LNP can improve the Th1 response induced by the SV, whereas LNP-CpG improves the Th1 response and alum improves the Th2 response.

### 3.3. SV plus LNP has Strong Protective Effects Against Influenza Virus Challenge

**Figure 5:**
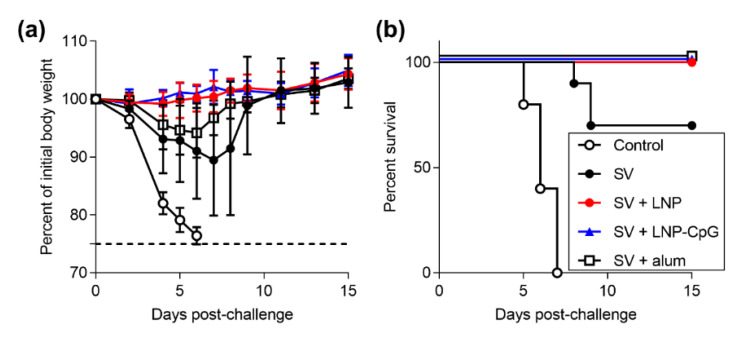
Protective effects against influenza virus challenge. Mice were subcutaneously immunized with the SV alone, SV plus LNP, SV plus LNP-CpG, or SV plus alum on day 0. On day 21, mice were challenged with Cal7. (**a**) Percentages of initial body weights and (**b**) survival rates were monitored after challenge with Cal7. *n* = 10. (**a**) Data are means ± SD.

To examine the protective effect of immunization with SV plus LNP, we challenged the immunized mice by using Cal7 at 21 days after the first immunization and observed their body weights (Figure 5a and Supplementary Figure S3a) and survival rates (Figure 5b). We observed rapid body weight loss in unimmunized control mice, and all mice died within seven days of the challenge. Mice immunized with the SV alone showed rapid weight loss, and 30% of them died within nine days of challenge. The groups immunized with SV plus LNP, SV plus LNP-CpG, or SV plus alum showed no decrease in survival rate. In addition, mice immunized with SV plus LNP or SV plus LNP-CpG showed no body weight loss, whereas mice immunized with SV plus alum showed a slight but significant body weight loss on day six after challenge compared to mice given SV plus LNP or SV plus LNP-CpG (Figure 5a, Supplementary Figure S3b). The data indicated that the immune responses induced by the LNP were sufficient to protect against virus challenge.

### 3.4. LNP Does Not Enhance Immune Responses When Used with Recombinant HA Protein as an Antigen

**Figure 6:**
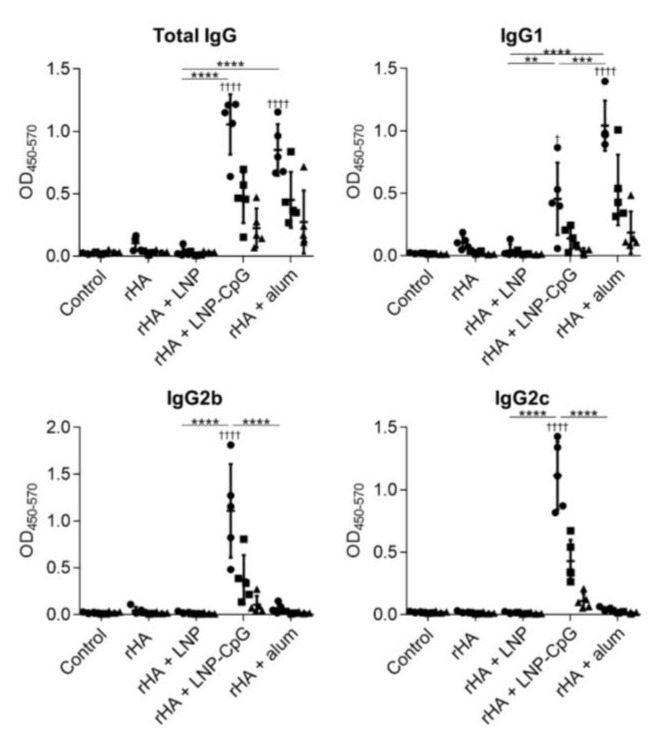
Hemagglutinin (HA)-specific antibody responses in vivo. Mice were subcutaneously immunized with recombinant HA (rHA) alone, rHA plus LNP, rHA plus LNP-CpG, or rHA plus alum on days 0 and 21. On day 28, the levels of rHA-specific total IgG, IgG1, IgG2b, and IgG2c in plasma were evaluated by using ELISA. We used 160- (●), 800- (■), and 4000- (▲) fold diluted plasma samples. *n* = 5. Data are means ± SD. Significant differences were only analyzed in the 160-fold-diluted plasma samples. ^†^ *p* < 0.05 and ^††††^ *p* < 0.0001 vs. group immunized with rHA alone; ** *p* < 0.01, *** *p* < 0.001, and **** *p* < 0.0001, as indicated by Tukey’s test.

**Figure 7:**
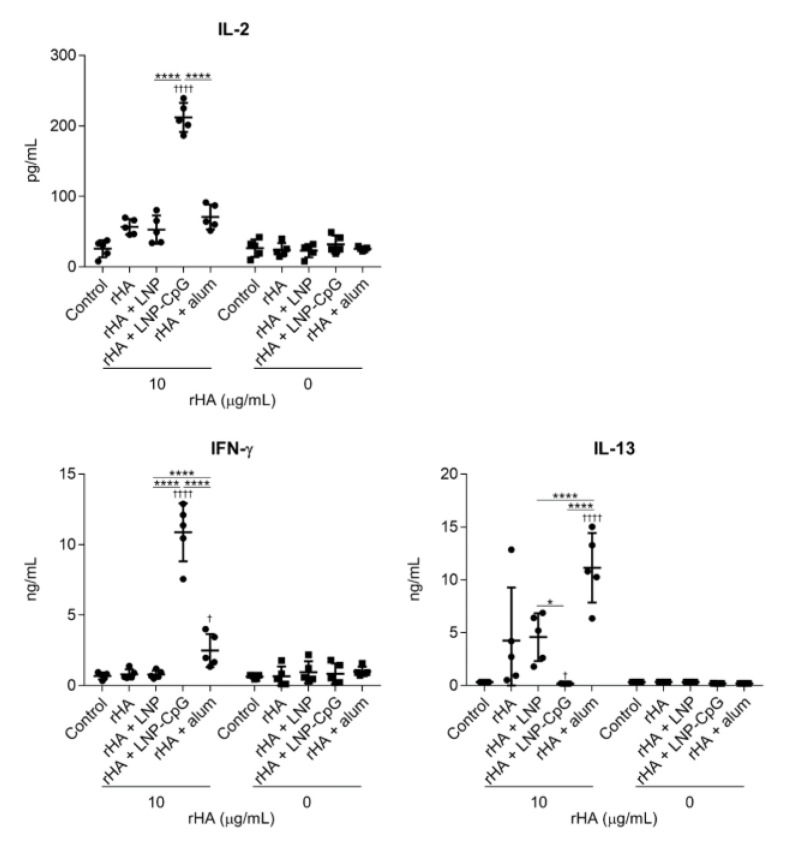
HA-specific T-cell responses in vivo. Mice were subcutaneously immunized with rHA alone, rHA plus LNP, rHA plus LNP-CpG, or rHA plus alum on days 0 and 21. On day 28, splenocytes were cultured in the presence or absence of rHA in vitro. After 1 (for IL-2) or 5 (for IFN-γ and IL-13) days, the levels of IL-2, IFN-γ, and IL-13 were measured by using ELISA. *n* = 5 per group. Data are means ± SD. ^†^ *p* < 0.05 and ^††††^ *p* < 0.0001 vs. group immunized with rHA alone; * *p* < 0.05, **** *p* < 0.0001, as indicated by Tukey’s test.

To further validate the results achieved with the SV, we evaluated the usefulness of the LNP with another antigen, namely the rHA protein. We used the ectodomain of HA from Cal7 to generate trimeric rHA. We immunized mice with rHA alone, rHA plus LNP, rHA plus LNP-CpG, or rHA plus alum, and we then measured the rHA-specific antibody responses (Figure 6) and T-cell responses (Figure 7). As was the case for immunization with the SV, the levels of rHA-specific total IgG and IgG1 induced by immunization with rHA plus LNP-CpG or with rHA plus alum were significantly higher than the level induced by immunization with rHA alone, whereas mice immunized with rHA plus alum showed no enhancement of rHA-specific IgG2b or IgG2c. In contrast, there were no significant differences in rHA-specific total IgG, IgG1, IgG2b, or IgG2c between mice given rHA alone and those given rHA plus LNP (Figure 6). In addition, the levels of IL-2, IL-13, and IFN-γ in the supernatant after splenocyte stimulation in mice immunized with rHA plus LNP were the same as those in mice given rHA alone, whereas we observed significantly higher levels of IL-2 and IFN-γ in mice immunized with rHA plus LNP-CpG, and of IFN-γ and IL-13 in mice given rHA plus alum, than in mice given rHA alone (Figure 7). These results suggested that the LNP does not enhance immune responses when rHA is used as an antigen.

### 3.5. LNP Does Not Induce Inflammatory Responses In Vivo

**Figure 8:**
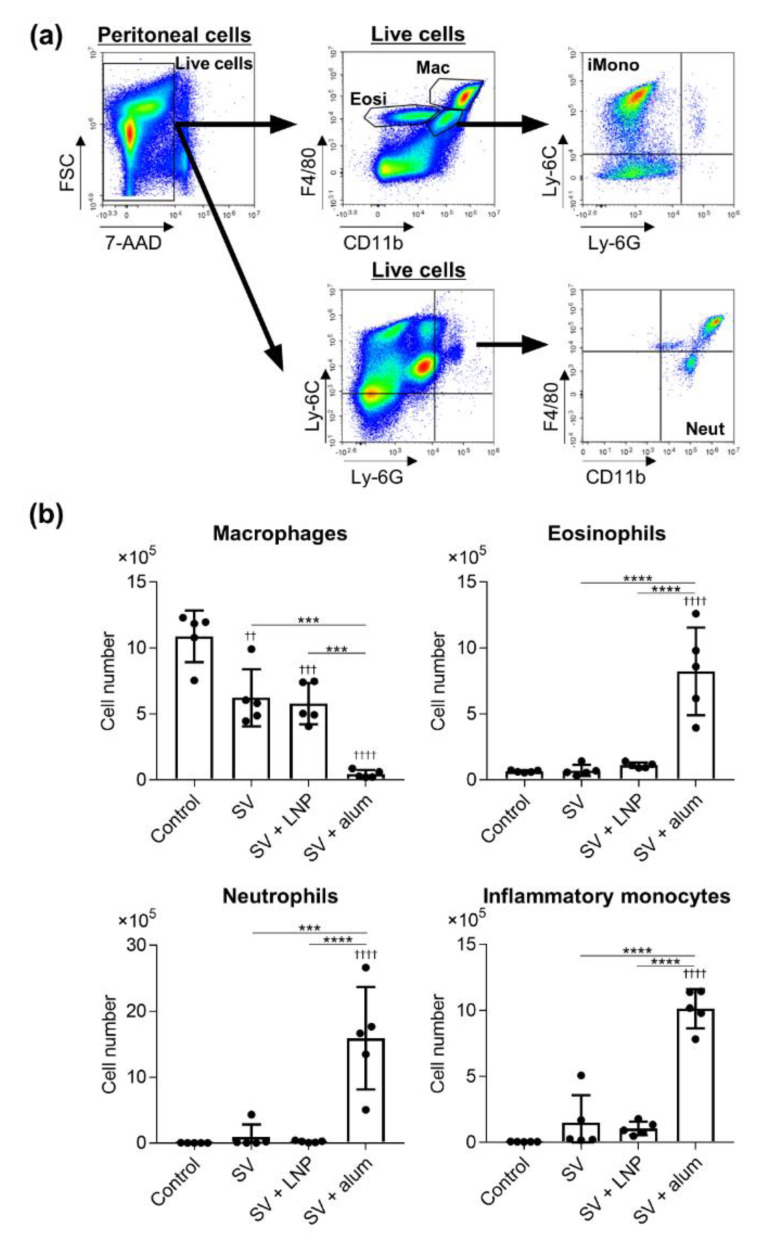
Recruitment of inflammatory immune cells by the LNP and alum in vivo. Mice were treated intraperitoneally with the SV alone, SV plus LNP, or SV plus alum. (**a**,**b**) After 20 h, the numbers of macrophages, eosinophils, neutrophils, and inflammatory monocytes in peritoneal lavage fluid were analyzed by flow cytometry. Gating schema for macrophages (Mac), eosinophils (Eosi), neutrophils (Neut), and inflammatory monocytes (iMono) are shown in (**a**). *n* = 5 per group. Data are means ± SD. ^††^ *p* < 0.01, ^†††^ *p* < 0.001, and ^††††^ *p* < 0.0001 vs. untreated control group; *** *p* < 0.001 and **** *p* < 0.0001, as indicated by Tukey’s test.

**Figure 9:**
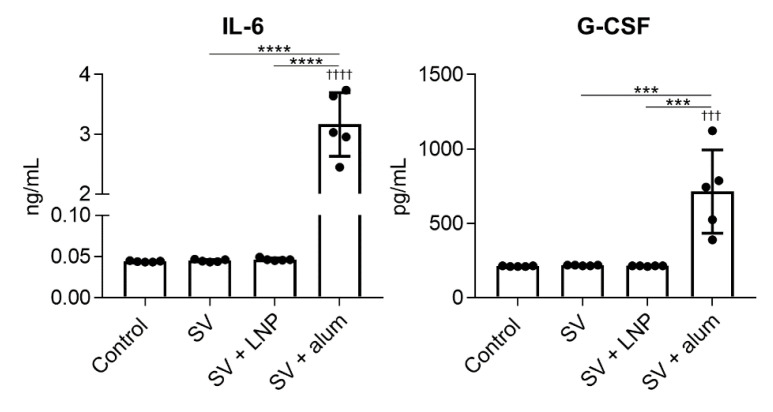
Production of inflammatory cytokines by the LNP and alum in vivo. Mice were treated intraperitoneally with the SV alone, SV plus LNP, or SV plus alum. After 4 h, the concentrations of IL-6 and granulocyte-colony stimulating factor (G-CSF) in peritoneal lavage fluid were measured by using ELISA. *n* = 5 per group. Data are means ± SD. ^†††^ *p* < 0.001 and ^††††^ *p* < 0.0001 vs. untreated control group; *** *p* < 0.001 and **** *p* < 0.0001, as indicated by Tukey’s test.

Alum induces inflammation, as indicated by the production of inflammatory cytokines and the recruitment of inflammatory immune cells at the site of immunization [30,31,32]. To compare the inflammatory responses between the LNP and alum, mice were treated with the SV alone, SV plus LNP, or SV plus alum intraperitoneally to facilitate sampling. Twenty hours after injection, we examined the numbers of macrophages, eosinophils, neutrophils, and inflammatory monocytes in peritoneal lavage fluid by using flow cytometry (Figure 8a,b). There was no difference in the number of macrophages between mice immunized with the SV alone and those given SV plus LNP, whereas the number of macrophages in mice treated with SV plus alum was significantly lower than those in mice given the SV alone or SV plus LNP (Figure 8b). In addition, the numbers of eosinophils, neutrophils, and inflammatory monocytes in mice treated with SV plus alum were significantly higher than those in mice given the SV alone or SV plus LNP, whereas we observed no differences in these numbers between mice given the SV alone and those given SV plus LNP (Figure 8b). Next, we examined the levels of IL-6 and G-CSF in peritoneal lavage fluid by using ELISA (Figure 9). Consistent with the results in Figure 8, there were no differences in the levels of IL-6 and G-CSF between mice immunized with the SV alone and those given SV plus LNP, whereas the levels of IL-6 and G-CSF in mice immunized with SV plus alum were significantly higher than those in mice given the SV alone or SV plus LNP. These results suggest that the LNP can induce SV-specific immune responses via a non-inflammatory pathway distinct from that induced by alum.

## 4. Discussion

Here, we showed that our LNP can improve SV-specific immune responses when the SV is used as an antigen (Figure 2, Figure 3 and Figure 4). Notably, we found that the LNP can induce SV-specific IgG1, IgG2b, IgG2c and Th1 responses, whereas alum can induce SV-specific IgG1, IgG2b, and Th2 responses (Figure 2, Figure 3 and Figure 4). In addition, we demonstrated that SV plus LNP can protect against influenza virus challenge as efficiently as SV plus alum (Figure 5), suggesting the usefulness of the LNP as an adjuvant for influenza vaccines. Our results support those of other recent studies that have highlighted the effectiveness of LNPs as vaccine adjuvants [23,24,25]. However, it is not clear whether our LNP can be used with other types of antigens because it does not enhance antigen-specific antibody responses and T-cell responses when rHA is used as an antigen (Figure 6 and Figure 7). In contrast, LNP-CpG and alum can strengthen rHA-specific antibody responses and T-cell responses (Figure 6 and Figure 7), indicating that the adjuvant mechanism of our LNP differs from those of LNP-CpG and alum. Therefore, we need to assess the versatility of our LNP for use with other antigens, and we also need investigate its adjuvant mechanism.

Recently, there has been a suggestion regarding part of the adjuvant mechanism of LNPs, although most of the mechanism remains unclear. Many LNPs, including our LNP, contain lipids with positive charges at physiological pHs, and these cationic lipids might contribute to the adjuvant activity of LNPs. In fact, many liposomes and LNPs formulated with cationic lipids are being developed as vaccine adjuvants [33,34,35]. In addition, Swaminathan et al. showed that reducing the cationic lipid content of their LNP reduced the LNP’s adjuvant effects [24]. Furthermore, some reports have shown that cationic lipids and cationic nanoparticles can activate TLR2, TLR4, or inflammasome pathways and can induce cytokine production by antigen-presenting cells [35,36,37]. Therefore, the activation of innate immunity by these pathways may be responsible for the adjuvanticity of LNPs, although there are no clear data to confirm the importance of these pathways. In contrast, we showed here that our LNP does not induce cytokine production by DCs or enhance the expression of co-stimulatory molecules on DCs (Figure 1), suggesting that our LNP does not activate innate immune responses. These results are consistent with our finding that our LNP could not enhance immune responses when rHA is used as an antigen (Figure 6 and Figure 7). The adjuvant mechanism of our LNP might differ from those of other LNPs, because the formulations of the LNPs that have been used thus far vary. Therefore, it is important to examine in future why our LNP improves immune responses when the SV is used as an antigen. For example, the interaction between our LNP and SV and between our LNP and rHA must be investigated, because a depot effect—the slow release of antigens—might be responsible for the adjuvanticity of our LNP. In addition, small nanoparticles (20 to 100 nm) are preferentially trafficked towards draining lymph nodes and taken up by DCs in the lymph nodes [38,39]. Therefore, our LNP with a diameter of 86.5 nm might have the ability to efficiently deliver the SV into draining lymph nodes via its interaction with the SV. Furthermore, by changing the formulation of the LNP, it might be possible to enhance the LNP’s interaction with not only the SV but also rHA, as well as to enhance the immune responses when rHA is used as an antigen. An understanding of the mode of action of our LNP will help in the successful design and development of LNPs as adjuvants.

We showed here that LNP-CpG induced SV-specific IgG2b, IgG2c (but not IgG1), and Th1 responses (Figure 2, Figure 3 and Figure 4). Consistent with these results, we previously showed the potential of the LNP as a CpG ODN delivery vehicle and the usefulness of LNP-CpG as an adjuvant for influenza vaccine when the SV was used as an antigen [27]. Though we did not examine whether the adjuvant effects of LNP-CpG in vivo depended on TLR9 in that study, our group showed in another report that cytokine production by bone marrow cells in vitro in response to LNP-CpG was completely dependent on TLR9 [26]. Taking these results together, we speculated that the adjuvant effects of LNP-CpG in vivo might be dependent on TLR9 in that study [27]. However, we speculate now that both TLR9-independent LNP-inducing immune responses and TLR9-dependent CpG-ODN-that induce innate immune activation contribute to the vaccine effects induced by LNP-CpG.

The use of adjuvants, such as TLR agonists and alum, with protein antigens is essential for enhancing vaccine effects in vivo. However, the unfavorable systemic inflammation caused in response to such adjuvants—especially TLR agonists—is typically responsible for adjuvant-dependent adverse reactions [10,40,41,42]. For example, one specific type of CpG ODN causes splenomegaly and disrupts splenic microarchitecture in mice after repeated administration [10,14]. In addition, poly(I:C), a TLR3 agonist, causes fever, erythema, and sometimes life-threatening endotoxin-like shock in humans [41]. Therefore, it is essential to investigate the inflammatory responses induced by adjuvants and reduce these responses. Recently, alum-induced cell death has been reported to be an important function of the adjuvanticity of alum [32,43]. Cell death results in the release of inflammatory molecules that result in the recruitment and activation of inflammatory cells [32,43]. In fact, the depletion of resident macrophages and the accumulation of neutrophils and eosinophils at the injection site have been reported in alum-treated mice [30,31,32]. Consistent with the results in Figure 1, we showed here that the LNP does not induce the depletion of peritoneal macrophages; the accumulation of neutrophils, eosinophils, and inflammatory monocytes; or inflammatory cytokine production, in contrast to alum in mice (Figure 8 and Figure 9); therefore, our LNP does not induce either DC activation in vitro or inflammatory responses in vivo. Therefore, we believe that our LNP might be a useful adjuvant platform that does not induce local inflammation when used with the influenza vaccine.

## 5. Conclusions

Here, we show the potential of the LNP as an adjuvant without inflammatory responses for influenza SVs. Our strategy should be useful for developing influenza vaccines with enhanced efficacy and safety.

## Supplementary Materials

The following are available online at https://www.mdpi.com/2076-393X/8/3/433/s1, Figure S1: Cytokine production by mouse-derived DCs in response to SV and LNP in vitro. Mouse-derived DCs were treated with SV (0.4 μg/mL), LNP (110 μg lipid/mL), or SV (0.4 μg/mL) plus LNP (110 μg lipid/mL) for 24 h in vitro. Levels of IL-6 and IL-12 p40 in the supernatants were measured by using ELISA. n = 5 per group. Data are means ± SD., Figure S2: Hemagglutinin (HA)- and neuraminidase (NA)-specific antibody responses in vivo. Mice were immunized subcutaneously with SV alone, SV plus LNP, SV plus LNP-CpG, or SV plus alum on days 0 and 21. On day 28, plasma levels of (a) recombinant HA-specific total IgG and (b) recombinant NA-specific total IgG were evaluated by using ELISA. (c) NA inhibition titers in plasma samples were evaluated by using ELLA. The two subpanels show the same data. The left subpanel shows percent inhibition relative to the plasma dilution. The right subpanel shows the 50% inhibitory concentrations (IC50 values) calculated from the curves in the left subpanel. (a–c) The same plasma samples as used in Figure 3 were used here. (a, b) We used 160- (●), 800- (■), and 4000- (▲) fold diluted plasma samples. *n* = 5 per group. Data are means ± SD. (a, b) Significant differences were analyzed only in the 160-fold-diluted plasma samples. ^†^ *p* < 0.05, ^††††^ *p* < 0.0001 vs. group immunized with SV alone; * *p* < 0.05, ** *p* < 0.01 as indicated by Tukey’s test, Figure S3: Protective effects against influenza virus challenge. Mice were immunized subcutaneously with SV alone, SV plus LNP, SV plus LNP-CpG, or SV plus alum on day 0. On day 21, mice were challenged with Cal7. (a) Percentages of initial body weight in individual mice were plotted. (b) Percentages of initial body weights on day 6 after Cal7 challenge were plotted. (a) n = 10. (b) *n* = 4 (Control), *n* = 10. (b) Data are means ± SD. ^†††^ *p* < 0.001 vs. group immunized with SV alone; * *p* < 0.05 (Tukey’s test) between indicated groups.
