# Supplementary material for: Lipid Nanoparticle Acts as a Potential Adjuvant for Influenza Split Vaccine without Inducing Inflammatory Responses
Source: Vaccines (Basel). 2020 Aug 3;8(3):433. doi: 10.3390/vaccines8030433 (PMC7565178; doi:10.3390/vaccines8030433)
Supplement: Supplementary file 1 [file vaccines-08-00433-s001.pdf]

vaccines

MDPI

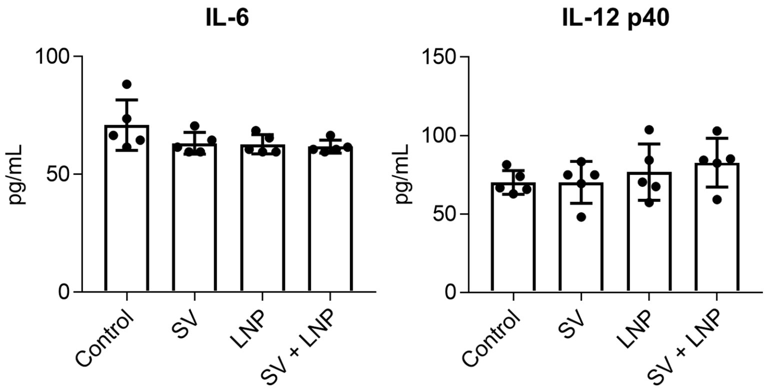

Figure S1: Cytokine production by mouse-derived DCs in response to SV and LNP in vitro. Mouse-derived DCs were treated with SV (0.4 μg/mL), LNP (110 μg lipid/mL), or SV (0.4 μg/mL) plus LNP (110 μg lipid/mL) for 24 h in vitro. Levels of IL-6 and IL-12 p40 in the supernatants were measured by using ELISA. *n* = 5 per group. Data are means ± SD.

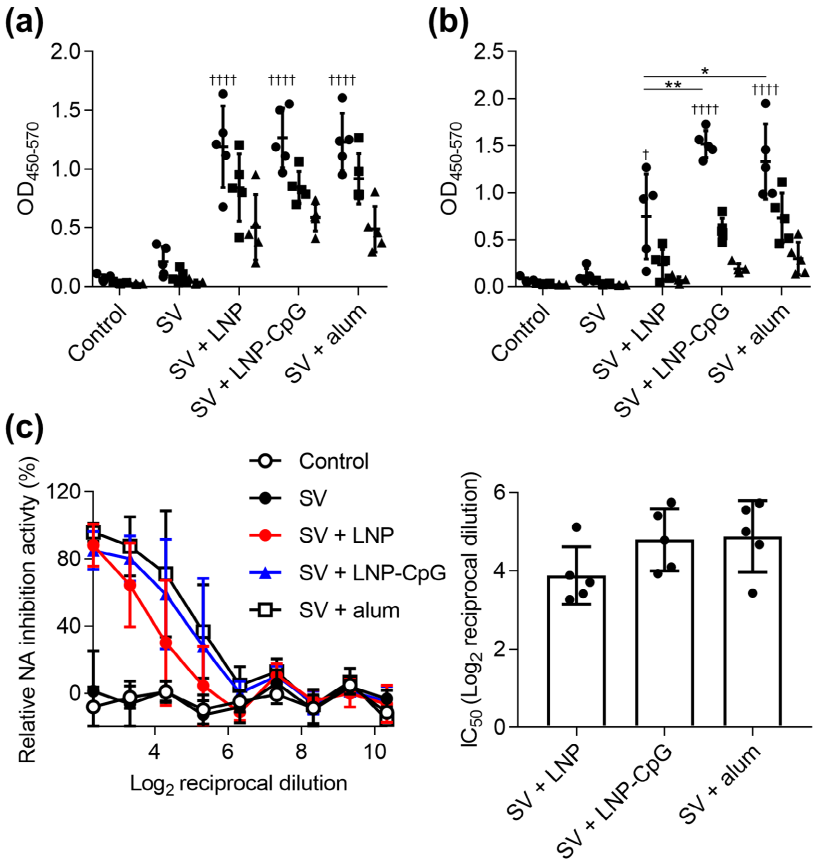

Figure S2: Hemagglutinin (HA)- and neuraminidase (NA)-specific antibody responses in vivo. Mice were immunized subcutaneously with SV alone, SV plus LNP, SV plus LNP-CpG, or SV plus alum on days 0 and 21. On day 28, plasma levels of (**a**) recombinant HA-specific total IgG and (**b**) recombinant NA-specific total IgG were evaluated by using ELISA. (**c**) NA inhibition titers in plasma samples were evaluated by using ELLA. The two subpanels show the same data. The left subpanel shows percent inhibition relative to the plasma dilution. The right subpanel shows the 50% inhibitory concentrations (IC50 values) calculated from the curves in the left subpanel. (a–c) The same plasma samples as used in Figure 3 were used here. (a, b) We used 160- (●), 800- (■), and 4000- (▲) fold diluted plasma samples. *n* = 5 per group. Data are means ± SD. (a, b) Significant differences were analyzed only in the 160-fold-diluted plasma samples. $^{\dagger}P < 0.05$, $^{\dagger\dagger\dagger\dagger}P < 0.0001$ vs. group immunized with SV alone; * $P < 0.05$, ** $P < 0.01$ as indicated by Tukey's test

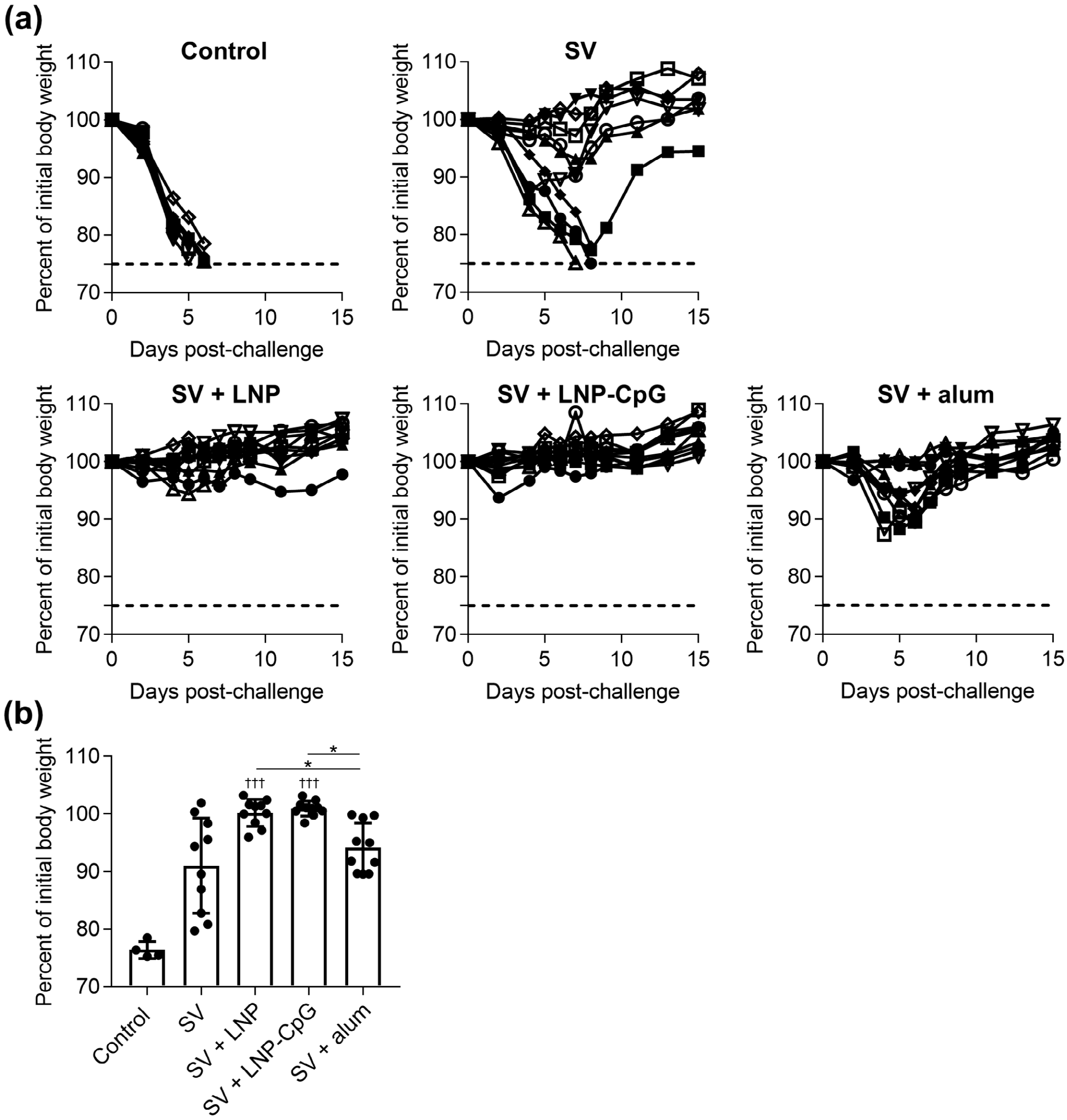

Figure S3: Protective effects against influenza virus challenge. Mice were immunized subcutaneously with SV alone, SV plus LNP, SV plus LNP-CpG, or SV plus alum on day 0. On day 21, mice were challenged with Cal7. (**a**) Percentages of initial body weight in individual mice were plotted. (**b**) Percentages of initial body weights on day 6 after Cal7 challenge were plotted. (a) n = 10. (b) *n* = 4 (Control), *n* = 10. (b) Data are means ± SD. ††† $P < 0.001$ vs. group immunized with SV alone; * $P < 0.05$ (Tukey's test) between indicated groups.
